# Epidemiological Profile of a Human Hepatitis E Virus Outbreak in 2018, Chattogram, Bangladesh

**DOI:** 10.3390/tropicalmed7080170

**Published:** 2022-08-06

**Authors:** Kei Owada, Joyantee Sarkar, Md. Kaisar Rahman, Shahneaz Ali Khan, Ariful Islam, Mohammad Mahmudul Hassan, Ricardo J. Soares Magalhães

**Affiliations:** 1 Queensland Alliance for One Health Sciences, School of Veterinary Science, The University of Queensland, Gatton, QLD 4343, Australia; 2 One Health Institute, Chattogram Veterinary and Animal Sciences University, Chattogram 4225, Bangladesh; 3 School of Veterinary Medicine, Texas Tech University, Amarillo, TX 79106, USA; 4 EcoHealth Alliance, New York, NY 10018, USA; 5 Faculty of Veterinary Medicine, Chattogram Veterinary and Animal Sciences University, Chattogram 4225, Bangladesh; 6 Children’s Health and Environment Program, UQ Children’s Health Research Centre, The University of Queensland, Brisbane, QLD 4072, Australia

**Keywords:** hepatitis E virus (HEV), outbreak investigation, spatial epidemiology, water source, climate, Bangladesh

## Abstract

Hepatitis E virus (HEV) is a waterborne zoonotic disease that can result in a high fatality rate in pregnant women and infants. In 2018, a large HEV outbreak emerged in Chattogram, Bangladesh, resulting in 2800 cases and a significant public health response to mitigate the transmission. While the source of the outbreak remained poorly understood, authorities suggested that possible risk factors for HEV infection included contamination of water supply, exacerbated by concurrent severe flooding events in the community. A cross-sectional study was conducted to investigate the distribution and risk factors for HEV seroprevalence between January and December 2018 in the Chattogram city area. A total of 505 blood samples were collected from symptomatic patients of 10 hospitals who met the case definition for an HEV infection. Standard ELISA tests were performed in all patients to identify anti-HEV antibodies. The size and location of HEV seroprevalence clusters within Chattogram were investigated using SaTScan. We investigated the association between risk of HEV infection and individual and environmentally lagged risk factors using Bernoulli generalised linear regression models. Our results indicate an overall HEV seroprevalence of 35% with significant variation according to sex, source of drinking water, and boiling of drinking water. A positive cross-correlation was found between HEV exposure and precipitation, modified normalised difference water index (MNDWI), and normalised difference vegetation index (NDVI). Our model indicated that risk of infection was associated with sex, age, source of drinking water, boiling of water, increased precipitation, and increased MNDWI. The results from this study indicate that source and boiling of drinking water and increased precipitation were critical drivers of the 2018 HEV outbreak. The communities at highest risk identified in our analyses should be targeted for investments in safe water infrastructure to reduce the likelihood of future HEV outbreaks in Chattogram.

## 1. Introduction

Hepatitis E virus (HEV) is a single-stranded RNA virus belonging to the genus *Orthohepevirus* in the *Hepeviridae* family. HEV infection is reported to be most common in east and south Asia [1] with seroprevalence estimated at 17–42% in southeast Asia [2] and above 30% among adults from India, Bangladesh, China, and Malaysia [3].

Early symptoms of HEV infection include mild fever, chills, headache, fatigue, and malaise, which are often associated with marked loss of appetite, aversion to food, upper abdominal discomfort, nausea, and vomiting. Evidence suggests that individuals with HEV infection often show no symptoms, and, during HEV outbreaks, asymptomatic individuals vastly outnumber symptomatic individuals [4]. HEV genotypes 1 and 2 are human viruses, while genotypes 3 and 4 are zoonotic, and the four HEV genotypes are known to cause acute liver failure and neurological illness. It is estimated that a total of 20 million infections with HEV genotypes 1 and 2 occur globally annually, with 3.4 million cases of symptomatic illness [5]. Genotypes 1 and 2 can also result in a high fatality rate in pregnant women and infants [4,6,7,8]. Pigs are the main zoonotic reservoir of genotypes 3 and 4 of HEV [3,8]. HEV genotype 3 infections in pigs have been detected in most parts of the world, including the Americas, Europe, Africa, Japan, southeast Asia, and Oceania, while genotype 4 has been primarily reported in pigs in China, Japan, and Indonesia [8].

The most common mode of transmission for HEV is the faecal–oral route; however, evidence points to some variation in the mode of transmission between the different genotypes, whereby genotypes 1 and 2 are primarily transmitted by contaminated water and contaminated food, and genotypes 3 and 4 primarily transmitted by consumption of undercooked meat and direct contact with infected animals [3]. Available evidence suggests that possible risk factors of HEV infection include socioeconomic status of affected populations [3,9], high urban population density in affected areas [10,11], and contamination of water supplies by human and animal waste, especially when exacerbated by seasonal weather events such as flooding [7,11,12,13]. Available evidence suggests that only HEV genotype 1 has been identified in large waterborne outbreaks in India, Nepal, Pakistan, and Bangladesh [3].

Bangladesh is an HEV hyperendemic country, having experienced major HEV waterborne outbreaks [3]. Previous cross-sectional studies in Bangladesh indicated HEV seroprevalence to range from 22.5% in rural areas to 60.1% in the capital of Dhaka [14]. Furthermore, a review of the current literature found five records of human HEV outbreaks in Bangladesh, which were all associated with contaminated drinking water: 2008–2009 in Dhaka [7], 2010 in Rajshahi City Corporation [11], 2013 in Noakhali [15], 2017 in Rajshahi City Corporation [15], and 2018 in Chattogram [15]. Evidence of seroprevalence of each HEV genotype in Bangladesh is limited [9,16]; however, the literature suggests outbreaks in Bangladesh have predominantly been due to genotypes 1 and 2 [3], with RNA testing during the 2010 outbreak in Rajshahi City detecting HEV genotype 1 [11].

The most recent HEV outbreak in Chattogram occurred between May and July 2018, coinciding with Bangladesh’s rainy season, and it affected approximately 2800 people in the Chattogram city area. Blood samples collected from suspected cases all tested positive for HEV; however, there are no data to confirm which genotype was the cause of the outbreak [15]. Anecdotal evidence suggested flooding and water supply contamination as being possible sources of this HEV outbreak [15,17,18]. Although contaminated drinking water was implicated in the origin of the 2018 outbreak, the Chittagong Water Supply and Sewerage Authority (CWASA) did not find any traces of HEV in the water. It was suggested that heavy rain and tidal water may have led to polluted water entering underground water reservoirs in the affected area in Chattogram [17]. The only study investigating the 2018 outbreak found an association between HEV infection and water source; however, it had a small sample size (case number = 92) and only sampled for 5 days in a localised geographical area [19]. This study did not examine the impact of environmental and climate-related factors such as rainfall and flooding on the outbreak. Furthermore, the spatial variation of the impact of the different HEV risk factors within Bangladesh has not been investigated to date.

A spatial epidemiological approach provides several valuable tools for understanding disease distribution, cluster detection, and geographical correlation. This approach has been used in other studies to investigate the effect of a wide range of risk factors of infectious diseases, including climate and environmental factors [20,21,22,23,24]. Spatial epidemiological approaches have been applied in previous studies to identify and characterise clusters of hepatitis infection, which could inform public health decisions and design spatially targeted control and prevention interventions that consider specific regional conditions [23,24].

The current study was initially set up in January 2018, before the 2018 HEV outbreak, to understand sporadic cases of HEV occurring in Chattogram in January that year. During the study, there was a sharp rise in HEV cases across Chattogram hospitals between April and June. Consequently, the current study extended its scope to encompass a comprehensive investigation of the epidemiological characteristics of HEV seroprevalence and risk factors during the 2018 HEV outbreak and to identify areas prone to HEV infection.

## 2. Materials and Methods

### 2.1. Ethics Approval

The study protocol was approved by the Ethics Committee of the Chattogram Veterinary and Animal Sciences University (CVASU), Bangladesh [permit reference number: CVASU/Dir (R&E) EC/2015/1011(03), Date: 27 December 2018].

### 2.2. Study Area

Chattogram is the second largest city of Bangladesh and located in the south-eastern part of Bangladesh with an area of 5282.98 km^2^ and located between the latitudes of 21°54′ and 22°59′ N and the longitudes of 91°17′ and 92°13′ E. Chattogram is subdivided into 15 upazilas (subdistricts) and Chattogram Metropolitan Area. The average monthly maximum temperature of the Chattogram district ranges from 26.0 °C to 32.4 °C, and the humidity ranges from 70% to 85%. The average monthly rainfall varies from 7.3 mm to 735.6 mm [25,26]. The population of Chattogram district is estimated to be 7,616,352 (2011 estimate), with an average population density of 1421 persons per km^2^. However, the population density of the capital Chattogram Metropolitan City is 16,677 persons per km^2^ [27].

### 2.3. Case Definition and Data Collection Procedures

Over the period of January to December 2018, suspected patients from sentinel sites in Chattogram were included in the study on the basis of fitting the case definition for an HEV case. In order to maximise efficiency in data collection, a purposive sampling method was applied. Of the 31 hospitals in Chattogram, a total of 10 hospitals with the highest general patient load were selected as sentinel sites. Hospitalised patients of any age were considered for selection on the basis of symptoms shown that matched the case definition of suspected HEV infection. The case definition considered the following symptoms: fever persisting for 2–10 days, headache, retro-orbital pain, myalgia, severe backache, rash, bleeding manifestations, abdominal pain, decreased urinary output despite adequate fluid intake, and irritability in infants [16].

Patients who presented to the 10 sentinel sites between January and December 2018 were invited to participate in this study, provide a blood sample, and complete a questionnaire [16]. The questionnaire collected information on demographics, household-level risk factors such as source of drinking water (deep tube well, shallow tube well, supplied water by Water Supply and Sewerage Authority (WASA)), boiling of water, and location of residence (Supplementary Table S1).

### 2.4. HEV Infection Data

Blood samples of patients were collected aseptically. All blood samples were tested for HEV using Wantai HEV-IgG, and Wantai HEV-IgM enzyme-linked immunosorbent assay (ELISA) kits [28], developed by Beijing Wantai Biological Pharmacy Enterprise Co., Ltd., Beijing, China [16]. Each patient’s test result was categorised as seropositive or seronegative, which was used as the outcome variable in the current study.

### 2.5. Remotely Sensed Environmental Data and Extraction

To evaluate any possible time lag effects in environmental data on HEV incidence, environmental data were retrieved for the period of 3 months prior to the estimated exposure date. This was to account for the seasonal effect in Bangladesh. The estimated exposure date was specified as 6 weeks before the test date for this analysis, according to the World Health Organisation estimate of the incubation period being 5–6 weeks on average [1]. The earliest test result was recorded on 2 January 2018, putting the earliest estimated exposure date at 21 November 2017. Therefore, environmental data were retrieved from August 2017 up until December 2018. All environmental data retrieved for analysis were averaged over the 8 day data period specified by the Moderate Resolution Imaging Spectroradiometer (MODIS) remote sensing data [29]. The environmental data used in the study are summarised in Table 1.

### 2.6. Data Analysis

#### 2.6.1. Descriptive Analyses

Pearson’s chi-squared test was performed using the *chisq.test()* function in the free statistical software R version 4.1.3 (R Core Team, Vienna, Austria) [36] to identify associations between HEV seroprevalence and each predictor. The predictors included sex (0 = male, 1 = female), age category in years (0 = 0–20, 1 = 21–40, 2 = 41–60, 3 = 61+), source of drinking water (0 = shallow tube well, 1 = deep tube well, 2 = supplied water by WASA), and boiling of water (0 = no, 1 = yes). Associations with *p* < 0.05 were considered statistically significant.

#### 2.6.2. Temporal Cross-Correlation between HEV Infection Incidence and Environmental Factors

HEV incidence and environmental data were aggregated to perform cross-correlation analysis. HEV incidence was aggregated into the incidence for each 8 day period. The spatial average of the environmental data was calculated over the combined area of the upazilas (subdistricts) containing the coordinates of the 505 individuals. The aggregated data were inputted into the *ccf()* function in R [36] to produce cross-correlation lag plots.

#### 2.6.3. Spatial Analysis Pipeline

We first visualised the HEV infection data by plotting the locations of individuals found positive and negative during the serosurvey using the mapping software ArcGIS version 10.8.1 (Environmental Systems Research Institute, Redlands, CA, USA) [34]. This was followed by a geographical exploration of the data by identifying the presence and size of HEV infection clusters in the study area. SaTScan version 10.0.2 (Kulldorff, M, Boston, MA, USA), which is free software that can detect spatial and temporal clusters in datasets using probabilistic models [37], was used to detect spatial clusters of HEV test results using a Bernoulli model. The maximum cluster size was reduced from the default of 50% of the dataset population to 25% due to the default setting detecting clusters that contained a majority of the study area. The clusters identified in SaTScan quantify the relative risk (RR) of HEV infection, which represents the risk of infection inside a cluster compared to outside of that cluster. The RR was calculated using the following formula:

(1) $$RR_{i}=\frac{P_{i}}{N_{i}\;}÷\frac{P_{TOTAL}-P_{i}}{N_{TOTAL}-N_{i}},$$

where *RR_i_* is the relative risk for cluster *i*, *P_i_* is the number of HEV-positive test results in cluster *i*, *N_i_* is the study population in cluster *i*, *P_TOTAL_* is the total number of HEV-positive test results in the study population, and *N_TOTAL_* is the study population of the entire study area. Pearson’s chi-squared test was used to evaluate association between HEV test results and month of test, demographics, and household predictors for each cluster.

We then modelled the associations between the risk of HEV infection and predictors. Each of these analyses is described in detail below. Prior to modelling, Spearman’s Rho correlation between risk of HEV infection and significant lags in environmental data, determined by cross-correlation analysis, was evaluated using the *corrMatrix()* function in the *jmv* package [38] in R [36]. Strong correlations were specified as those with an absolute rho value of 0.8 or greater. Such strong correlations were analysed separately to avoid collinearity between environmental data. A Bernoulli generalised linear model (GLM) was created using the *glm* command in the statistical analysis software Stata version 13.1 (StataCorp, College Station, TX, USA) [39] using HEV test result as the outcome variable, whereas sex, age category, source of drinking water, boiling of drinking water, environmental variables with significant lags, distance to water bodies, and elevation were used as predictors. Variables with a *p*-value of ≤0.2 were included in the final GLM. Variables in the final GLM with a *p*-value of <0.05 were considered statistically significant. Correlation between source and boiling of drinking water was also investigated using the *corr* command in Stata [39], where an output of ±1 represents perfect correlation and 0 represents no correlation.

Lastly, using the residuals from the final Bernoulli GLM, semivariance was calculated as a function of the max distance of half the maximum separation distance between coordinates in the dataset, and a semivariogram was generated using this semivariance result in R [36] to assess the presence of residual spatial autocorrelation in the dataset. Due to many patients residing in identical coordinates (i.e., apartment blocks), the *jitterDupCoords()* function was used to apply a random offset of up to 0.001 degrees (~100 m). The semivariogram is defined by three parameters: the nugget (*y*-value at *x* = 0), the sill (maximum semivariance value), and the range (the lag distance where the sill is reached). The difference in semivariance between the sill and the nugget is called the partial sill [40]. If the resulting semivariogram shows an exponential shape with values increasing with distance, this would indicate the existence of spatial autocorrelation [40]. Propensity of clustering was determined by calculating the percentage of the semivariogram that is due to clustering using the following formula:

(2) $$Propensity\;of\;clustering=\frac{Partial\;Sill}{Nugget+Partial\;Sill}.$$

## 3. Results

### 3.1. Descriptive Analyses

**Figure 1:**
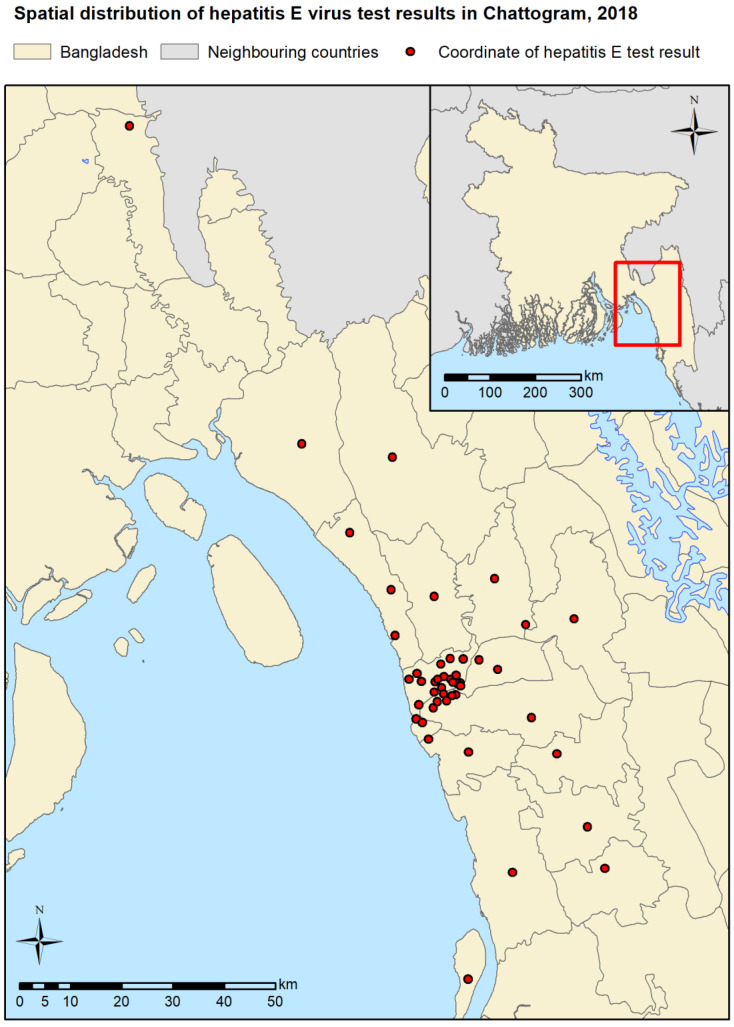
Map of spatial distribution of hepatitis E virus (HEV) test results in study area. Some coordinates contain multiple test results.

A total of 516 patients presenting to the 10 participating hospitals fitted our case definition. Of these, 505 patients agreed to provide a blood sample and complete a questionnaire for the current study, producing a study dropout rate of 2.13% (Figure 1). Of the 505 patients, 177 (35.05%) tested seropositive for HEV, and 328 (64.95%) tested seronegative. Most patients were aged 21–40 years (55.45%) and were male (56.83%). The most common reported drinking water source was “shallow tube well” (42.97%), followed by “WASA” (37.03%) and “deep tube well” (20.00%). Most individuals (83.17%) did not boil their drinking water regardless of water source.

Our results (Table 2) indicate significant associations between HEV-positive serology and sex, source of drinking water, and boiling of water (*p* < 0.05), in that a higher proportion of cases were found in males compared to females (41.11% compared to 27.06%), in those with WASA compared to those with tube well water sources (60.96% compared to 26.73% and 16.59%), and in those that did not report boiling water prior to consumption (40.95% compared to 5.88%).

### 3.2. Temporal Variation in HEV Infection and Associated Risk Factors

**Figure 2:**
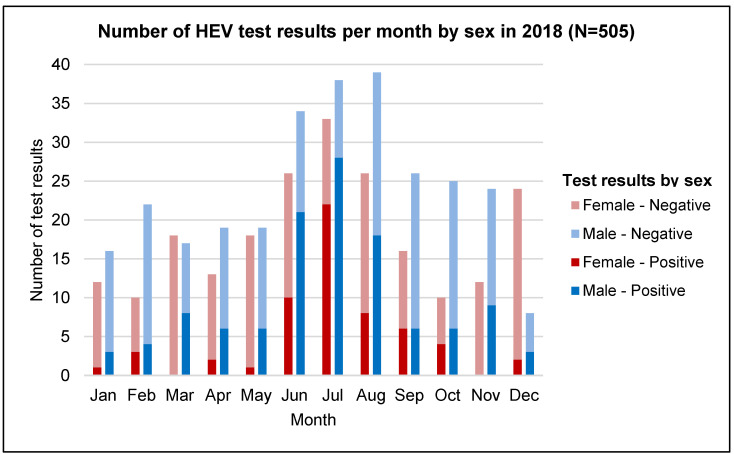
Number of HEV test results per month by sex in 2018. There are three major seasons in Bangladesh—summer (March to June), rainy season (July to October), and winter (November to February).

In 2018, HEV-seropositive test results for both males and females showed a significant increase between June and August, peaking in July (Figure 2). This coincides with the rainy season, which occurs during the months of July to October. In all months, there were more seropositive test results for males than females.

**Figure 3:**
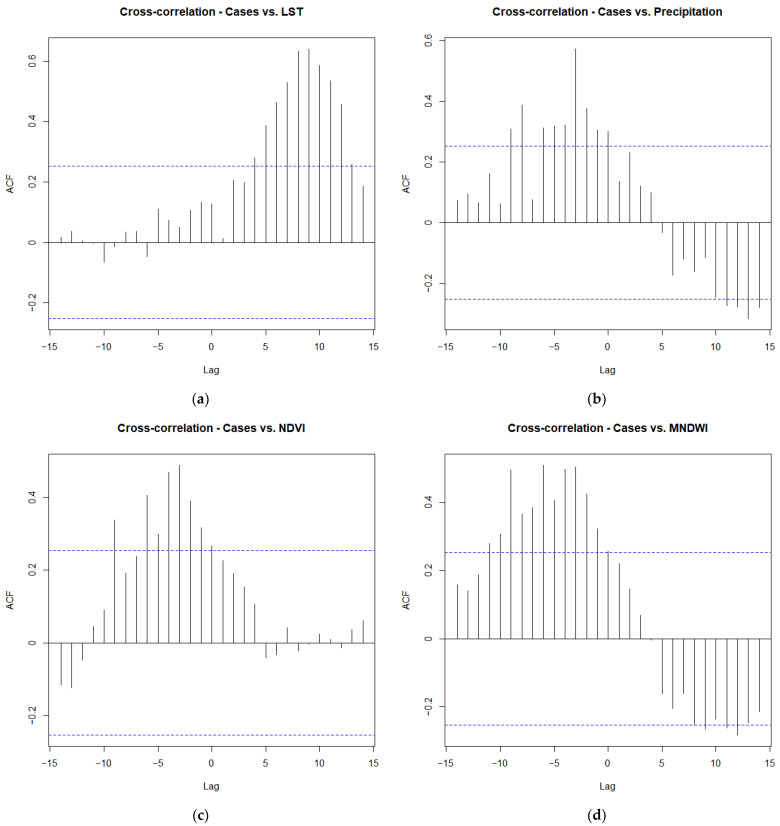
Cross-correlation time lag effects between HEV cases and (**a**) LST, (**b**) precipitation, (**c**) NDVI, and (**d**) MNDWI.

A maximum significant and positive cross-correlation was found between HEV exposure and precipitation (0.574), modified normalised difference water index (MNDWI) (0.504), and normalised difference vegetation index (NDVI) (0.489), all at a lag of 3 (24 days) prior to exposure. However, no significant cross-correlation was found between HEV exposure and land surface temperature (LST) (Figure 3). This suggests that an increase in any of precipitation, MNDWI, and NDVI 24 days prior to exposure may lead to an increased incidence of HEV.

### 3.3. Spatial Analyses

**Figure 4:**
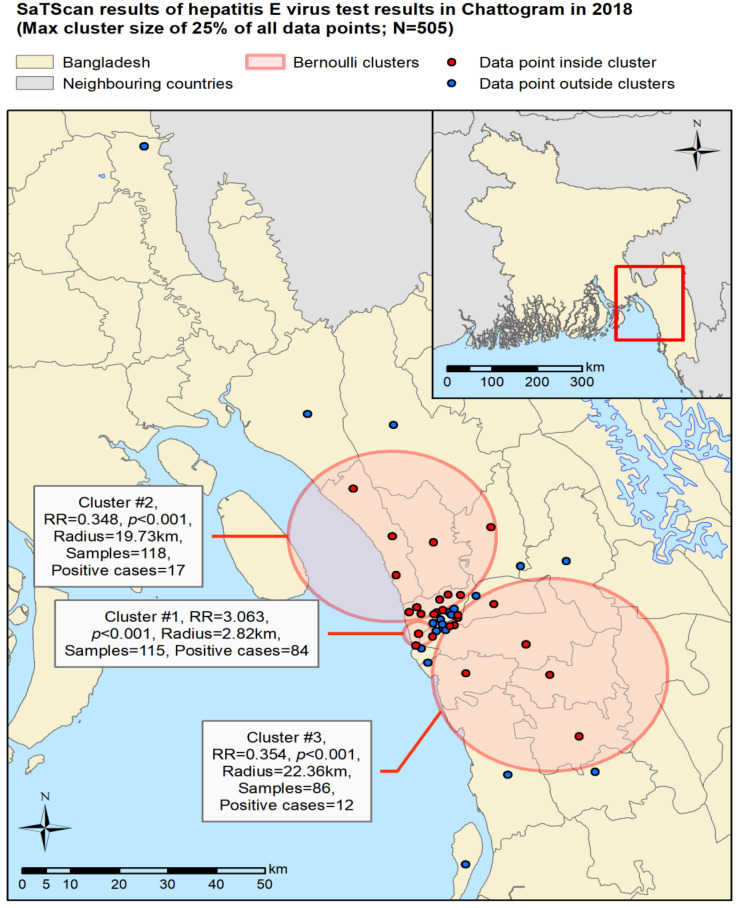
SaTScan clusters for HEV test results in Bangladesh, where RR represents relative risk and *p* represents *p*-value.

A total of three statistically significant (*p* < 0.001) clusters of HEV seroprevalence were identified in the study area, ranging in radius from 2.83 km to 22.36 km (Figure 4). Cluster 1 had the smallest radius; however, it was found to have a much higher RR of 3.063, compared to the RR of 0.348 and 0.354 in clusters 2 and 3, respectively (Figure 4). For cluster 1, statistically significant associations were found between HEV test result and test month (*p* < 0.01), drinking water source (*p* < 0.05), and boiling of drinking water (*p* < 0.01). For cluster 2, a statistically significant association was found between test result and sex (*p* < 0.05) and boiling of drinking water (*p* < 0.05). For cluster 3, a statistically significant association was found between test result and age category (*p* < 0.01).

The correlation function between drinking water source and boiling of water was evaluated as 0.238, suggesting weak correlation; therefore, no interaction term between these two predictors was included in the Bernoulli GLM. Furthermore, a strong correlation (rho ≥ |0.8|) was found between NDVI and MNDWI, indicating high collinearity between these two variables. Of these two environmental data types, NDVI was discarded from the Bernoulli GLM in favour of MNDWI, because MNDWI is an indicator of heavy rainfall and flooding (NDVI is an indicator of vegetation) [20], and flooding has been found to be associated with increased HEV incidence [12].

Our results of the final multivariable model indicate that the probability of HEV infection was statistically significant and positively associated with the 41–60 years age group (*p* = 0.007), deep tube well (*p* = 0.009) and WASA (*p* < 0.001) drinking water sources, precipitation with a lag of 1 (8-days) (*p* < 0.001), and MNDWI with a lag of 5 (40-days) (*p* = 0.022) (Table 3). Our results also indicate a significant and negative association between the probability of HEV infection and females (*p* = 0.001), boiling of drinking water (*p* < 0.001), and precipitation with a lag of 6 (*p* < 0.001) (Table 3).

**Figure 5:**
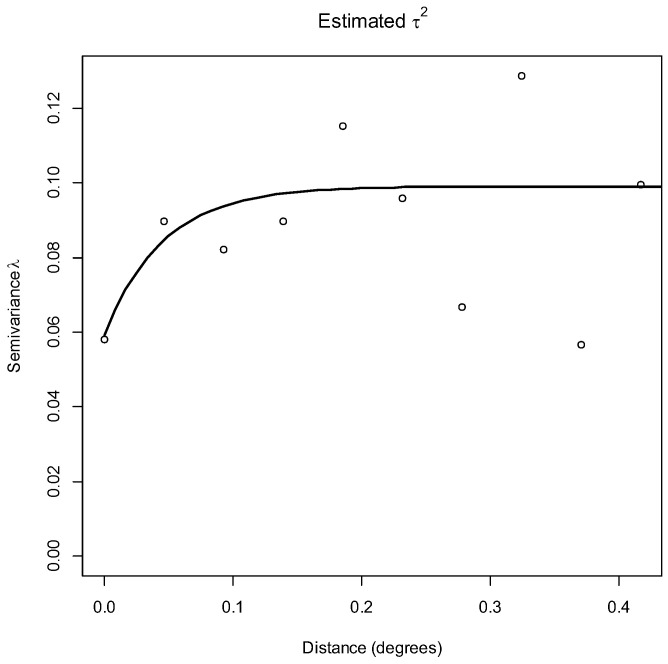
Semivariogram showing autocorrelation at 15.21 km.

The residual semivariogram showed spatial autocorrelation in HEV test results at a distance of 15.21 km, with a 40.24% propensity for clustering (Figure 5).

## 4. Discussion

### 4.1. Overall Findings

To our knowledge, the current study is the first to document up-to-date spatial and temporal variation of HEV seroprevalence in humans in the Chattogram district of Bangladesh.

Our study presents a novel spatial epidemiological approach to investigating possible risk factors of HEV infection using data collected from sentinel sites in Chattogram district in Bangladesh by accounting for the unique climate and environmental conditions such as land surface temperature, precipitation, and elevation, along with individual and household-level characteristics.

Our study found significant spatial variation in HEV seroprevalence associated with sex, age, drinking water source, boiling of water, and the climate (precipitation and MNDWI) of the study area in Bangladesh, with an overall seroprevalence of 35%. This supports the anecdotal evidence that the HEV outbreak in Chattogram may have resulted from rainfall and flooding leading to water supply contamination [15,17,18].

Our study is the first to present a sentinel surveillance-based approach to analysing the 2018 HEV outbreak in Chattogram. Previous studies have shown that sentinel surveillance can assist in understanding the trend of diseases over time in the broader area by using a representative sample of the population [41,42]. The findings from the 10 sentinel sites selected to monitor the incidence of HEV with respect to the temporal and spatial variation of risk factors for environmentally driven zoonosis may help in identifying the communities most at risk in the wider Chattogram area, as well as help in the design and implementation of targeted HEV prevention and control interventions.

### 4.2. Association between Risk of HEV Infection and Demographics

Our final Bernoulli GLM found that females were less at risk of HEV infection than males. Previous studies in both rural and urban Bangladesh areas found lower seroprevalence in females compared to males, and they suggested thatwomen may be less exposed to potential sources of HEV due to limited mobility [9,43]. Furthermore, only cluster 2 (radius 19.73 km) identified in our SaTScan analysis (Figure 4) found a significant association between HEV seroprevalence and sex. This cluster contained several households in areas outside of Chattogram city, which may have an increased differential in mobility between men and women than in the city. This highlights the need to examine contamination of water supply, not just at the household level, but also in the wider community [43].

In our final Bernoulli GLM, the age group of 41–60 years was found to be more at risk of HEV infection compared to those aged 0–20 years. Available evidence suggests that this is a predominant age group for pig owners in parts of Bangladesh [44]. Another study found that the level of knowledge regarding disease prevention and biosecurity practices is low among animal owners in selected areas of Bangladesh, including Chattogram [45]. While this evidence regarding exposure to possible animal reservoirs of zoonotic HEV may explain the increased risk of HEV infection among this age group, the relationship between age and HEV infection in the current study context is unclear.

### 4.3. Association between Risk of HEV Infection and Drinking Water Source and Boiling of Water

Our study found a statistically significant association between risk of HEV infection and the use of WASA as drinking water source, and this may indicate that WASA water was contaminated with HEV. Furthermore, a statistically significant association between risk of HEV infection and drinking water source was also found within a localised geographical area centred in Halishahar (Figure 4) which is a densely populated area within Chattogram city where 55.1% of the population uses CWASA, according to the 2011 national census [27]. Our SaTScan results determined this area to have the highest RR of HEV infection in the study area. This suggests that people in densely populated areas, where drinking of contaminated WASA water is common, may be at increased risk of HEV infection. A statistically significant association was also found between risk of HEV infection and not boiling drinking water. Haque et al. (2015) investigated an HEV outbreak recorded in Rajshahi city in northern Bangladesh in 2010 and found drinking of municipal tap water to be a risk factor of HEV infection [11]. This study suggested that raising awareness about boiling of drinking water could reduce the risk of waterborne diseases. The findings of the current study are in accordance with this suggestion.

In addition, precipitation was found to have a statistically significant association with risk of HEV infection. Combined with the findings on source of drinking water, these two factors may suggest that flooding due to increased rainfall may be causing contaminated water, such as sewage, and runoff from animal facilities, such as animal manure and wastewater [12], to enter the WASA network.

One study analysed water supply quality by collecting random samples from four routes of the CWASA distribution system. Microbial water quality was found to deteriorate as it flowed through the distribution system, with a lack of separation between supply lines and sewers identified as one of the reasons for this [13]. Previous studies found contaminated water sources to be the leading risk factor during HEV outbreaks in Bangladesh in 2008–2009 [7] and 2010 [11], and that water contamination is an ongoing problem in various locations in Bangladesh. Contamination of water supplies by animal reservoirs may be considered as a possible risk factor for HEV infection in humans; however, the current study did not test the water supply for the presence of HEV. Further studies could perform HEV RNA testing on the drinking water at HEV patients’ residences to determine the possible sources of contamination [12].

### 4.4. Association between Risk of HEV Infection and Climate

Precipitation and MNDWI were all found to play a significant role in risk of HEV infection, with a common maximum lag effect at 24 days prior to the estimated exposure date according to cross-correlation time lag analysis. Furthermore, after adjusting for the effect of sex, age, source of drinking water, and boiling of drinking water in our final Bernoulli GLM, increased precipitation 8 days prior to exposure and increased MNDWI 40 days prior to exposure were found to increase the risk of HEV infection. This confirms that heavy rainfall and flooding affect the risk of HEV exposure, which might suggest that contaminated water entered the drinking water network following a flood event, leading to exposure to HEV in households. A previous study indicated that the geographical distribution of HEV outbreaks due to HEV genotypes 1 and 2 could be predicted by precipitation seasonality, and that flood events may lead to faecal contamination of water sources [10]. In the current study, genetic testing was not performed. Therefore, further studies are required to identify whether the source of contamination originates from human waste or waste from animal reservoirs.

The World Bank projects that annual rainfall in Bangladesh will significantly increase in the coming decades (74 mm between 2040 and 2059) and that increased rainfall will intensify the spread of infectious diseases [46]. This highlights the importance of our findings related to the impact of precipitation on the spatial distribution of risk of HEV infection in Chattogram and its neighbouring areas in Bangladesh.

### 4.5. Possible Confounding Risk Factors of HEV Infection

The residual semivariogram showed spatial autocorrelation in HEV test results at a distance of 15.21 km (Figure 5) with a 40.24% propensity for clustering. This suggests that the GLM model does not fully account for second-order spatial variation, and that other factors may be influencing spatial autocorrelation to differing degrees at this distance. These factors could include individual behaviour including open defecation practices, sanitation and hygiene practices, sewage system infrastructural vulnerabilities, and distribution of animal HEV hosts in the local area. Previous studies have shown that poor hygiene, low sanitation, and open defecation practices are associated with waterborne HEV outbreaks [7,10,47]. Furthermore, it has been hypothesised that if HEV were to be excreted by urine then it could be transmitted by aerosols [48]. Further studies could include these factors in surveys to better understand the associations between risk of HEV infection and risk factors at the household level.

Presently the exact pig population in Bangladesh is not known; however, it has been reported as increasing in tribal areas as a means of improving livelihood [44]. A recent review on HEV in pig farms identified risk factors, including contaminated manure storage, drinking water, and fomites, such as feeding and drinking equipment [49]. Recent studies conducted in Bangladesh found overall low knowledge among animal owners of disease prevention and poor biosecurity practices such as not using disinfectants and having no waste management system [44,45]. This allows HEV to persist in pigs, which may in turn contribute to continued environmental contamination. Although the current study did not investigate ownership or contact with animal reservoirs, this could be another possible confounding factor of risk of HEV infection. Zoonotic HEV has been detected in pigs in various countries [50]. HEV genotype 4 has been identified in pigs in India [3], and HEV infection has been detected in pigs and pig handlers in Bangladesh, indicating possible zoonotic HEV transmission [47,51]; however, these studies did not specify which genotypes of HEV were detected. This suggests that it may be beneficial if future epidemiological studies of HEV infection take a One Health approach, whereby investigations include pig ownership and HEV genotype variations, as well as if pigs have been imported from regions where zoonotic HEV infection has been recorded, such as India. Identifying the genotype of an HEV infection could lead to the identification of the HEV reservoir, which in turn could help with targeting of both human and zoonotic HEV prevention and control strategies in Bangladesh.

### 4.6. Limitations

Our findings may be influenced by the quality of the environmental data used in the analysis. LST data of sufficient spatial and temporal resolution were only available from remote sensing data sources. Daytime LST data from MODIS on Nasa’s Terra satellite [52] were initially chosen to provide LST data for Bangladesh; however, it was observed that there were substantial spatial gaps in the data during times of high cloud cover. This was particularly evident during the rainy season months of June through to August. Alternate satellite data were searched, but all suffered from the same problem during times of high cloud cover. Two different approaches to interpolating the gaps in the data were explored [30,53]. Of these, the approach taken by Zhang et al. (2022) provided the most complete coverage of the study area over the entirety of the required time range [30]. They claimed an average root mean squared error of 1.88 °C in their dataset [30]. This may have affected the results of cross-correlation and Bernoulli GLM analyses performed in the current study. Second, the absence of certain predictor variables may have affected the results of our Bernoulli GLM analysis. These include spatial distribution of the pig population in Bangladesh and survey answers relating to animal ownership, individual hygiene practices, and knowledge of zoonotic disease prevention and control. Our study was only able to collect data from 10 of the 31 hospitals in Chattogram due to limited funding to support further data collection at the remaining hospitals. Our study used purposive sampling at 10 hospitals (of a total of 31 currently functioning hospitals), representing those hospitals with the highest patient load in Chattogram. This decision was made with the intent to capture a sample as representative as possible of the entire Chattogram area. Lastly, RNA testing for HEV genotype in humans, animals, and drinking water may also have assisted in identifying the source of the HEV outbreak.

## 5. Conclusions

The results from this study highlighted that source and boiling of drinking water and increased precipitation were critical drivers of the 2018 HEV outbreak in Chattogram. The analyses in our study identified the communities at the highest risk of HEV infection. These communities should be targeted for investments in safe water infrastructure to reduce the likelihood of future HEV outbreaks in Chattogram.

## Figures and Tables

**Table 1:** List of environmental data used in the study.

Environmental Variable	Resolution	Temporal Range	Source
Land surface temperature (LST)	1 km	Daily	[30]
Precipitation	0.1 degrees	Daily	[31]
Elevation	0.00083 degrees	N/A	[32]
Inland water bodies ^1^	Vector	N/A	[33]
Modified normalised difference water index (MNDWI) ^2^	500 m	8 day average	[29]
Normalised difference vegetation index (NDVI) ^2^	500 m	8 day average	[29]

^1^ Distance to inland water bodies was calculated in ArcGIS version 10.8.1 (Environmental Systems Research Institute, Redlands, CA, USA) [34]. ^2^ MNDWI and NDVI were calculated on the basis of the Moderate Resolution Imaging Spectroradiometer (MODIS) reflectance data using previously established methods [20,35].

**Table 2:** Summary of HEV survey results and associated Pearson’s chi-squared test results.

Variable	Category	HEV Test Result			Proportion of All Patients (*N* = 505)	Pearson’s Chi-Squared Test
		Seronegative	Seropositive	Total		
Age	0–20	59 (68.60%)	27 (31.40%)	86	17.03%	chi^2^ = 4.78, *p* = 0.189
	21–40	182 (65.00%)	98 (35.00%)	280	55.45%	
	41–60	65 (58.56%)	46 (41.44%)	111	21.98%	
	61+	22 (78.57%)	6 (21.43%)	28	5.54%	
Sex	Male	169 (58.89%)	118 (41.11%)	287	56.83%	chi^2^ = 10.74, *p* = 0.001
	Female	159 (72.94%)	59 (27.06%)	218	43.17%	
Source of drinking water	Shallow tube well	181 (83.41%)	36 (16.59%)	217	42.97%	chi^2^ = 90.71, *p* < 0.001
	Deep tube well	74 (73.27%)	27 (26.73%)	101	20.00%	
	WASA	73 (39.04%)	114 (60.96%)	187	37.03%	
Boiling of water	No	248 (59.05%)	172 (40.95%)	420	83.17%	chi^2^ = 38.19, *p* < 0.001
	Yes	80 (94.12%)	5 (5.88%)	85	16.83%	

**Table 3:** Results of the final multivariable Bernoulli GLM of associations between HEV infection and demographic, contextual and environmental factors.

Variable	Predictor	Coefficient	Standard Error	*p*-Value	95% Confidence Interval
Sex (reference: male)	Female	−0.118	0.034	0.001	(−0.186, −0.051)
Age category (reference: 0–20 years)	21−40	0.035	0.047	0.458	(−0.057, 0.127)
	41−60	0.146	0.055	0.007	(0.039, 0.253)
	60+	−0.108	0.083	0.190	(−0.271, 0.054)
Source of drinking water (reference: shallow tube well)	Deep tube well	0.124	0.047	0.009	(0.032, 0.216)
	WASA	0.444	0.047	0.000	(0.353, 0.535)
Boiling of drinking water (reference: no)	Yes	−0.504	0.047	0.000	(−0.596, −0.412)
Environment time Lag effect	Precipitation (lag 8 days)	0.086	0.023	0.000	(0.041, 0.131)
	Precipitation (lag 48 days)	−0.098	0.024	0.000	(−0.145, −0.051)
	Precipitation (lag 72 days)	0.022	0.018	0.206	(−0.012, 0.057)
	MNDWI (lag 40 days)	0.049	0.021	0.022	(0.007, 0.091)
	LST (lag 8 days)	0.029	0.020	0.155	(−0.011, 0.069)
Intercept		0.235	0.049	0.000	(0.140, 0.330)

## Data Availability

The data presented in this study are available on request from the corresponding author. The data are not publicly available due to privacy reasons.

## Supplementary Materials

The following supporting information can be downloaded at https://www.mdpi.com/article/10.3390/tropicalmed7080170/s1: Supplementary Table S1. Metadata of human HEV samples collected during the outbreak in 2018, Chattogram, Bangladesh.

## References

[1] World Health Organization (2021). Hepatitis E. https://www.who.int/news-room/fact-sheets/detail/hepatitis-e.

[2] Paul W., Supharerk T., Thammasin I., Wikrom K. (2020). Hepatitis E in Southeast Asia. Siriraj Hosp. Gaz..

[3] Wang Y. (2016). Hepatitis E Virus.

[4] World Health Organization (2014). Waterborne Outbreaks of Hepatitis E: Recognition, Investigation and Control: Technical Report. https://www.who.int/publications-detail-redirect/9789241507608.

[5] Rein D.B., Stevens G.A., Theaker J., Wittenborn J.S., Wiersma S.T. (2012). The global burden of hepatitis E virus genotypes 1 and 2 in 2005. Hepatology.

[6] Borkakoti J., Hazam R.K., Mohammad A., Kumar A., Kar P. (2013). Does high viral load of hepatitis E virus influence the severity and prognosis of acute liver failure during pregnancy?. J. Med. Virol..

[7] Gurley E.S., Hossain M.J., Paul R.C., Sazzad H.M.S., Islam M.S., Parveen S., Faruque L.I., Husain M., Ara K., Jahan Y. (2014). Outbreak of Hepatitis E in Urban Bangladesh Resulting in Maternal and Perinatal Mortality. Clin. Infect. Dis..

[8] Denner J. (2019). Hepatitis E virus (HEV)-The future. Viruses.

[9] Labrique A.B., Zaman K., Hossain Z., Saha P., Yunus M., Hossain A., Ticehurst J., Nelson K.E. (2009). Population Seroprevalence of Hepatitis E Virus Antibodies in Rural Bangladesh. Am. J. Trop. Med. Hyg..

[10] Carratalà A., Joost S. (2019). Population density and water balance influence the global occurrence of hepatitis E epidemics. Sci. Rep..

[11] Haque F., Banu S.S., Ara K., Chowdhury I.A., Chowdhury S.A., Kamili S., Rahman M., Luby S.P. (2015). An outbreak of hepatitis E in an urban area of Bangladesh. J. Viral Hepat..

[12] Yugo D.M., Meng X.J. (2013). Hepatitis E virus: Foodborne, waterborne and zoonotic transmission. Int. J. Environ. Res. Public Health.

[13] Zuthi M.F.R., Biwas M., Bahar M.N. (2009). Assessment of supply water quality in the Chittagong city of Bangladesh. ARPN J. Eng. Appl. Sci..

[14] Kmush B., Wierzba T., Krain L., Nelson K., Labrique A.B. (2013). Epidemiology of Hepatitis E in Low- and Middle-Income Countries of Asia and Africa. Semin. Liver Dis..

[15] Khan M.H. (2018). Hepatitis E: Major Outbreaks in Bangladesh. https://iedcr.gov.bd/nbph/issue-sections/1ff3b5d9-35dd-4bb3-bb71-33797ea1b5f4.

[16] Sarkar J. (2019). Seroprevalence of Hepatitis-E Virus Infection among Patients Attending Different Hospitals at Chattogram, 2018. Master’s Thesis.

[17] Hussain A. (2018). Hepatitis E Outbreak in Chittagong City. https://archive.dhakatribune.com/health/2018/06/27/hepatitis-e-outbreak-in-chittagong-city.

[18] Daily Sun (2018). Hepatitis E Breaks Out in Port City. https://www.daily-sun.com/printversion/details/318535/Hepatitis-E-breaksout-in-port-city.

[19] Alam H.M., Maruf A., Khan M.H., Billah M.M., Uzzaman M.S., Flora M.S. Hepatitis outbreak in Halishahor, Chattagram, Bangladesh. Proceedings of the 9th Southeast Asia & Western Pacific Bi-regional TEPHINET Scientific Conference.

[20] Dhewantara P.W., Hu W., Zhang W., Yin W.-W., Ding F., Mamun A.A., Soares Magalhães R.J. (2019). Climate variability, satellite-derived physical environmental data and human leptospirosis: A retrospective ecological study in China. Environ. Res..

[21] Kirby R.S., Delmelle E., Eberth J.M. (2017). Advances in spatial epidemiology and geographic information systems. Ann. Epidemiol..

[22] Gao L., Zhang Y., Ding G., Liu Q., Jiang B. (2016). Identifying flood-related infectious diseases in Anhui Province, China: A spatial and temporal analysis. Am. J. Trop. Med. Hyg..

[23] Stopka T.J., Goulart M.A., Meyers D.J., Hutcheson M., Barton K., Onofrey S., Church D., Donahue A., Chui K.K.H. (2017). Identifying and characterizing hepatitis C virus hotspots in Massachusetts: A spatial epidemiological approach. BMC Infect. Dis..

[24] Zhu B., Liu J., Fu Y., Zhang B., Mao Y. (2018). Spatio-temporal epidemiology of viral hepatitis in China (2003-2015): Implications for prevention and control policies. Int. J. Environ. Res. Public Health.

[25] Khatun M.A., Rashid M.B., Hygen H.O. (2018). Climate of Bangladesh. http://bmd.gov.bd/file/2016/08/17/pdf/21827.pdf.

[26] Bangladesh Meteorological Department (2018). Normal Monthly Humidty. http://bmd.gov.bd/file/2016/08/17/pdf/21827.pdf.

[27] Bangladesh Bureau of Statistics (2014). Bangladesh Population and Housing Census 2011-National Report Volume-03: Urban Area Report. http://www.bbs.gov.bd/site/page/47856ad0-7e1c-4aab-bd78-892733bc06eb/Population-and-Housing-Census.

[28] Beijing Wantai Biological Pharmacy Enterprise Co., Ltd. (2020). Hepatitis E Virus Markers ELISAs. https://www.ystwt.cn/wp-content/uploads/2018/04/Wantai-HEV-IgG-ELISA.pdf.

[29] Vermote E. (2021). MODIS/Terra Surface Reflectance 8-Day L3 Global 500m SIN Grid V061. https://lpdaac.usgs.gov/products/mod09a1v061/.

[30] Zhang T., Zhou Y., Zhu Z., Li X., Asrar G.R. (2022). A global seamless 1 km resolution daily land surface temperature dataset (2003–2020). Earth Syst. Sci. Data.

[31] Huffman G.J., Stocker E.F., Bolvin D.T., Nelkin E.J., Tan J. (2020). Integrated Multi-satellitE Retrievals for GPM (IMERG), Version 06B. https://arthurhouhttps.pps.eosdis.nasa.gov/gpmdata/YYYY/MM/DD/gis/.

[32] Jarvis A., Reuter H.I., Nelson A., Guevara E. (2008). Hole-filled Seamless SRTM Data V4. https://srtm.csi.cgiar.org.

[33] Hijmans R. (2022). DIVA-GIS. https://www.diva-gis.org/.

[34] Environmental Systems Research Institute (2020). ArcGIS Desktop: Release 10.8.1. https://www.esri.com.

[35] Xu H. (2006). Modification of normalised difference water index (NDWI) to enhance open water features in remotely sensed imagery. Int. J. Remote Sens..

[36] R Core Team (2021). R: A Language and Environment for Statistical Computing. https://www.R-project.org/.

[37] Kulldorf M. (2022). SaTScan™ v10.0.2: Software for the Spatial and Space-Time Scan Statistics. https://www.satscan.org.

[38] Selker R., Love J., Dropmann F., Moreno V. (2022). jmv R Package (version 2.3.4). https://www.jamovi.org/jmv/.

[39] StataCorp (2013). Stata Statistical Software: Release 13. https://www.stata.com.

[40] Pfeiffer D., Robinson T.P., Stevenson M., Stevens K.B., Rogers D.J., Clements A.C.A. (2008). Chapter 6: Spatial variation in risk. Spatial Analysis in Epidemiology.

[41] Talaat M., Afifi S., Reaves E.J., Abu Elsood H., El-Gohary A., Refaey S., Hammad R., Abdel Fadeel M., Kandeel A. (2019). Evidence of sustained reductions in the relative risk of acute hepatitis B and C virus infections, and the increasing burden of hepatitis a virus infection in Egypt: Comparison of sentinel acute viral hepatitis surveillance results, 2001–2017. BMC Infect. Dis..

[42] Woyessa A.B., Mengesha M., Belay D., Tayachew A., Ayele W., Beyene B., Kassa W., Zemelak E., Demissie G., Amare B. (2018). Epidemiology of influenza in Ethiopia: Findings from influenza sentinel surveillance and respiratory infection outbreak investigations, 2009–2015. BMC Infect. Dis..

[43] Azman A.S., Paul K.K., Bhuiyan T.R., Koyuncu A., Salje H., Qadri F., Gurley E.S. (2021). Hepatitis E in Bangladesh: Insights From a National Serosurvey. J. Infect. Dis..

[44] Anower A.K.M., Ahmed M., Rahman M.M., Hasan A., Islam M.A., Rahman L. (2017). Hygienic Farming System Improved Pig-Rearers Livelihood Status in South-West Region of Bangladesh. Int. J. Avian Wildl. Biol..

[45] Nath T.C., Eom K.S., Choe S., Islam S., Sabuj S.S., Saha E., Tuhin R.H., Ndosi B.A., Kang Y., Kim S. (2022). Insights to helminth infections in food and companion animals in Bangladesh: Occurrence and risk profiling. Parasite Epidemiol. Control.

[46] The World Bank (2021). Climate Change in Bangladesh: Impact on Infectious Diseases and Mental Health. https://www.worldbank.org/en/news/feature/2021/10/07/climate-change-in-bangladesh-impact-on-infectious-diseases-and-mental-health.

[47] Nahar N., Uddin M., Sarkar R.A., Gurley E.S., Khan M.S.U., Hossain M.J., Sultana R., Luby S.P. (2013). Exploring pig raising in Bangladesh: Implications for public health interventions. Vet. Ital..

[48] Van der Poel W.H.M. (2014). Food and environmental routes of Hepatitis E virus transmission. Curr. Opin. Virol..

[49] Meester M., Tobias T.J., Bouwknegt M., Kusters N.E., Stegeman J.A., van der Poel W.H.M. (2021). Infection dynamics and persistence of hepatitis E virus on pig farms-a review. Porcine Health Manag..

[50] Harrison L.C., DiCaprio E. (2018). Hepatitis E Virus: An Emerging Foodborne Pathogen. Front. Sustain. Food Syst..

[51] Haider N., Khan M.S.U., Hossain M.B., Sazzad H.M.S., Rahman M.Z., Ahmed F., Zeidner N.S. (2017). Serological evidence of hepatitis E virus infection in pigs and jaundice among pig handlers in Bangladesh. Zoonoses Public Health.

[52] Wan Z., Hook S., Hulley G. (2021). MODIS/Terra Land Surface Temperature/Emissivity 8-Day L3 Global 1 km SIN Grid V061. https://lpdaac.usgs.gov/products/mod11a2v061/.

[53] Shiff S., Helman D., Lensky I.M. (2021). Worldwide continuous gap-filled MODIS land surface temperature dataset. Sci. Data.

